# Prevalence, incidence, and mortality rates of breast cancer in Kazakhstan: data from the Unified National Electronic Health System, 2014–2019

**DOI:** 10.3389/fpubh.2023.1132742

**Published:** 2023-04-17

**Authors:** Anna Midlenko, Kamilla Mussina, Gulnur Zhakhina, Yesbolat Sakko, Gyunel Rashidova, Bolat Saktashev, Dauren Adilbay, Oxana Shatkovskaya, Abduzhappar Gaipov

**Affiliations:** ^1^Department of Surgery, School of Medicine, Nazarbayev University, Astana, Kazakhstan; ^2^Department of Medicine, School of Medicine, Nazarbayev University, Astana, Kazakhstan; ^3^School of Sciences and Humanities, Nazarbayev University, Astana, Kazakhstan; ^4^Department of Mammology, Oncological Center of Tomotherapy “UMIT”, Astana, Kazakhstan; ^5^Department of Otolaryngology Head and Neck Surgery, Louisiana State University Health Sciences, Shreveport, LA, United States; ^6^Department of Scientific and Strategic Activities, Kazakh Research Institute of Oncology and Radiology, Almaty, Kazakhstan; ^7^Clinical Academic Department of Internal Medicine, CF “University Medical Center”, Astana, Kazakhstan

**Keywords:** breast cancer, incidence, mortality, prevalence, Kazakhstan breast cancer, Kazakhstan

## Abstract

**Background:**

Although there are numerous sources of epidemiologic information on breast cancer in Kazakhstan, none of them have specifically examined the burden of this disease. Therefore, this article aims to provide an overview of the breast cancer prevalence, incidence, mortality, and distribution and changes over time in Kazakhstan based on nationwide large-scale healthcare data from the National Registry in order to encourage more research on the impact of various diseases at the regional and national levels.

**Methods:**

The study cohort included all adult women older than 25 years who were diagnosed with breast cancer in any clinical setting of the Republic of Kazakhstan during the period of 2014–2019. The data were extracted from the Unified Nationwide Electronic Health System (UNEHS) to get an overview of descriptive statistics, incidence, prevalence, and mortality rate calculations and the Cox proportional hazards regression model. All survival functions and factors associated with mortality were tested for significance.

**Results:**

The cohort population (*n* = 55,465) comprised subjects with the age at the diagnosis of breast cancer from 25 to 97 years, with a mean of 55.7 ± 12.0 years. The majority of the study population belonged to the age group 45–59 years, which is 44.8% of the cohort. The all-cause mortality rate of the cohort is 16%. The prevalence rate increased from 30.4 per 10,000 population in 2014 to 50.6 in 2019. The incidence rate varied from 4.5 per 10,000 population in 2015 to 7.3 in 2016. Mortality rates were stable and high in the senile age patients (75–89 years old). Breast cancer mortality was positively associated with women who had been diagnosed with diabetes, HR 1.2 (95% CI, 1.1–2.3), whereas it was negatively associated with arterial hypertension, HR 0.4 (95% CI, 0.4–0.5).

**Conclusion:**

Overall, Kazakhstan is experiencing an increase in the incidence of breast cancer cases, but the mortality rate has started to decline. The switch to population mammography screening could reduce the breast cancer mortality rate. These findings should be utilized to help Kazakhstan determine what cancer control priorities should be utilized, including the need to implement efficient and affordable screening and prevention programs.

## 1. Introduction

Breast cancer is one of the most commonly seen malignant diseases among the female population worldwide, represents the most commonly diagnosed type of cancer, and was the main reason for cancer-related death in 2020 (1, 2).

Overall, the number of cancer incidences and mortality rates are rapidly growing worldwide. There was an estimated rate of 19 million new breast cancer cases and approximately 10 million cancer deaths worldwide in 2020, and there is an estimated rate of breast cancer death of ~11 million by 2030 (3, 4).

Breast cancer is the foremost and most commonly diagnosed type of cancer, which is closely followed by lung, colorectal, prostate, and stomach cancers (5, 6). Breast cancer ranks first for its incidence in a majority of countries and is the fifth highest cause of cancer-related mortality globally, accounting for one in four cancer diagnoses in female individuals and one in six cancer-related deaths (4).

Breast cancer incidence is significantly higher in developed countries than in developing countries (55.9 and 29.7 per 100,000, respectively), with the highest incidence rates registered in Australia and New Zealand, Western and Northern Europe, and North America and the lowest rates in Central America, Eastern and Middle Africa, and South-Central Asia. The mortality rates among women in developed countries are also 17% higher than those among women in developing countries (15.0 and 12.8 per 100 thousand, respectively) (2, 4, 7). Nevertheless, incidence rates of breast cancer have been rising in the last few years in developing countries, where rates were historically very low (5, 8, 9). Breast cancer incidence in developing countries demonstrates low numbers in comparison with developed countries because there is an insufficiency of data and no effective cancer registry, and the age-standardized incidence rates are estimated based on data from other countries and hospital registries (10).

Breast cancer incidence in the former Soviet Union countries was generally lower in comparison with European countries, with age-standardized rates ranging from 19.5 cases per 100 thousand population in Tajikistan to 57.5 in Georgia in 2020. At the same time, the mortality rate is considered to be similar to and higher than that in European countries (11).

The majority of the former Soviet Union countries have mammography-based health screening programs but are typically provided on an as-needed basis with insufficient quality, according to the WHO Country Capacity Survey 2019 (12). In most countries that offer regular mammography screening, the target age of the screened population is from 50 to 69 years old, but in some countries, such as Georgia, Kazakhstan, and Russian Federation, it also covers women who are 40–49 years old and younger (13).

Kazakhstan is a former Soviet Union republic with a population of 19 million and with almost 11 million urban population. The median age in Kazakhstan is 30.7 years with 78 years of life expectancy at birth in female patients and 69.6 years in male patients. There are nearly 4.6 thousand new cases of breast cancer and 1.3 thousand deaths registered in Kazakhstan annually; however, this report is limited to demographic, regional, and cause-specific associations with prevalence, incidence, and mortality rates (14).

Breast cancer epidemiological studies enable the development of program efficiency indicators and the evaluation of program implementation outcomes. They also enable the planning of screening and diagnostic activities targeted at early disease identification (14, 15).

Epidemiological data on breast cancer in Kazakhstan are available from different resources; however, there is not enough publication examining the prevalence of breast cancer in Kazakhstan. There are only five studies on breast cancer epidemiology in Kazakhstan published on PubMed from 2012 to 2021, and data were retrieved from regional and national cancer centers and cancer registries. Those studies provide information on cancer statistics but do not show any correlation with comorbidities.

This study aims to provide an overview of breast cancer prevalence, incidence, mortality, and distribution and changes over time in Kazakhstan based on the Unified Nationwide Electronic Health System (UNEHS). The registry includes both inpatient and outpatient registries. The main information for epidemiological investigation is patient's demographics, data on morbidity and mortality, comorbidities, complications, and medical procedures. Analysis from this database will assess epidemiological data on breast cancer in Kazakhstan and the impact of various diseases at the regional level on breast cancer outcomes. Additionally, the results of this study will help to identify possible opportunities for improvement of local public health when comorbidities are taken into account in the future. It may lead to optimal patient care with improved breast cancer patient outcomes.

## 2. Methods

### 2.1. Study design and settings

The data were retrieved from the “Electronic Registry of Inpatients”, which is one of the parts of the Unified Nationwide Electronic Health System (UNEHS), which was introduced at the end of 2013 to synchronize health data storage across the nation's healthcare system (16).

The registry includes both inpatient and outpatient registries. The main information for epidemiological investigation is patient's demographics, data on morbidity and mortality, comorbidities, complications, and medical procedures. Every patient is assigned a unique life-long population registry number (RpnID). RpnID uniquely identifies each citizen within any registry of UNEHS, and it is used to perform data linkage between different registries and for the creation of the main outcome variable—all-cause mortality. All diagnoses are coded by the International Classification of Diseases, Tenth Revision (ICD-10) (C50.0–C.50.6 for breast cancer). ICD-10 codes corresponding to breast cancer disease are used for studying the epidemiology of disease by the calculation of prevalence and incidence along with being analyzed as explanatory variables for outcomes.

### 2.2. Study population

**Figure 1:**
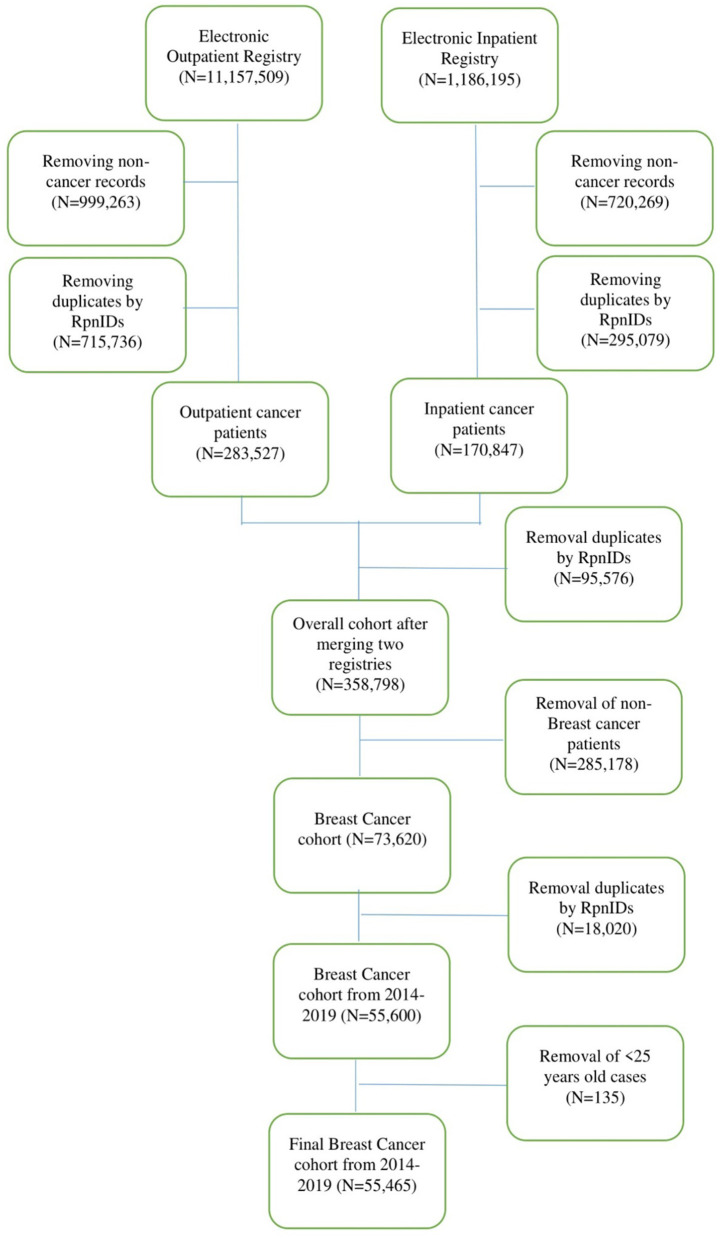
Flow chart diagram of cohort set-up.

The study population consisted of patients with breast cancer in any clinical setting of the Republic of Kazakhstan during the period from 2014 to 2019. The cohort included all adult women older than 25 years. All-cause mortality was taken from patients with breast cancer. The data cleaning and management procedures are represented in the flow chart (Figure 1).

### 2.3. Statistical analyses

Data analysis was performed using Stata MP2 16.1 version. Descriptive statistics were performed to characterize the socio-demographic and medical characteristics of the study population. Mean and standard deviation were described as continuous variables with normal distribution, and frequencies and proportions were determined as categorical variables. Two-sided *t*-tests for parametric and Mann–Whitney *U*-tests for nonparametric data were performed to determine the difference between groups. The proportions were determined by Pearson's chi-square tests or a Fisher exact test.

The Cox proportional hazards (PH) regression analysis was performed to estimate unadjusted and adjusted socio-demographic hazard functions for the prediction of survival probabilities of breast cancer patients as well as the investigation of associations with other risk factors such as hypertension, diabetes, and stroke. The magnitude of hazard ratios (HR) and the width of their 95% confidence intervals (CI) were considered in order to decide whether associations are statistically and clinically significant.

Cox regression analyses were performed to demonstrate crude and adjusted hazard ratios. A total of three multivariable analysis models were constructed to test the adjusted effect of variables on mortality. The models were adjusted for potential confounders depending on the theoretical background and their availability in the database. In the first model, unadjusted socio-demographic predictors (age, ethnicity, and residence) were included. In the second model, variables were adjusted to demographics. In the third model, comorbidities such as arterial hypertension, diabetes, and stroke were added to Model 2. In all models, the stepwise selection method was used. The fit of the models was evaluated by the Akaike information criterion, Bayesian information criterion, and global goodness-of-fit test. The statistical significance level was set at a *p*-value of 0.05.

## 3. Results

During the study period, 55,465 cases of breast cancer were registered from 2014 to 2019. The socio-demographic and some medical characteristics of the patients are presented in Table 1. The age at diagnosis of breast cancer cases ranges from 25 to 97 years with a mean age of 55.7 ± 12.0 years. The majority of the study population is the age group 45–59 years, which is 44.8 % of the cohort. The most represented ethnic groups were Kazakh (40.8%), followed by other ethnic groups (39.5%). Almost all cohorts (94.6%) are urban, and 98.5% of patients were hospitalized as planned. All-cause mortality for the cohort is 16%. The median follow-up time was 4.1 years (IQR, 2.0–9.0 years). Crude death rate (per 1,000 people) revealed that older age groups, Russian ethnic groups, rural residents, and urgent hospital admissions are considered risk factors for breast cancer mortality. Non-communicable diseases such as hypertension, diabetes, and stroke were taken as comorbidities. Data analysis showed that the majority of patients had hypertension (33.7%).

**Table 1:** Socio-demographic and medical characteristics of patients with breast cancer.

**Demographic characteristics**	**Total *n* = 55,465**	**Alive *n* = 46,336 (84%)**	**Dead *n* = 9,129 (16%)**	***p*-value**	**Mortality rate per 1,000 PY [95% CI]**
**Age, mean** ±**SD**	55.7 ± 12.0	54.8 ± 11.6	59.8 ± 13.2	<0.0001	
**Age groups**, ***n*** **(%)**				<0.0001	
25–44	10,089 (18.2)	8,903 (88.2)	1,186 (11.8)		12.7 [12.0; 13.4]
45–59	24,861 (44.8)	21,521 (86.6)	3,340 (13.4)		19.0 [18.4; 19.6]
60–74	16,684 (30.1)	13,534 (81.1)	3,150 (18.9)		36.8 [35.5; 38.1]
75–89	3,741 (6.7)	2,332 (62.3)	1,409 (37.7)		96.9 [92.0; 102.2]
>90	90 (0.2)	46 (51.1)	44 (48.9)		192.9 [143.5; 259.2]
**Ethnicity**, ***n*** **(%)**				<0.0001	
Other	21,858 (39.5)	18,727 (85.7)	3,131 (14.3)		23.8 [23.0; 24.7]
Kazakh	22,591 (40.8)	18,572 (82.2)	4,019 (17.8)		24.3 [23.6; 25.1]
Russian	10,867 (19.7)	8,908 (82.0)	1,959 (18.0)		27.1 [25.9; 28.3]
**Residence**, ***n*** **(%)**				<0.0001	
Rural	3,017 (5.4)	2,796 (92.7)	221 (7.3)		30.1 [26.4; 34.3]
Urban	52,448 (94.6)	43,540 (83.0)	8,908 (17.0)		24.6 [24.1; 25.1]
**Hospital admission**, ***n*** **(%)**				<0.0001	
Planned	22,215 (98.5)	19,115 (86.0)	3,100 (14.0)		48.5 [46.9; 50.3]
Urgent	327 (1.5)	159 (48.6)	168 (51.4)		156.4 [134.4; 181.9]
**Comorbidities**, ***n*** **(%)**					
Hypertension	18,719 (33.7)	16,452 (87.9)	2,267 (12.1)	<0.0001	15.8 [15.2; 16.5]
Diabetes	6,250 (11.3)	5,022 (80.4)	1,228 (19.6)	<0.0001	25.6 [24.2; 27.1]
Stroke	1,037 (1.9)	615 (59.3)	422 (40.7)	<0.0001	51.1 [46.4; 56.2]
Surgeries	7,175 (12.9)	6,755 (94.1)	420 (5.9)	<0.0001	

**Figure 2:**
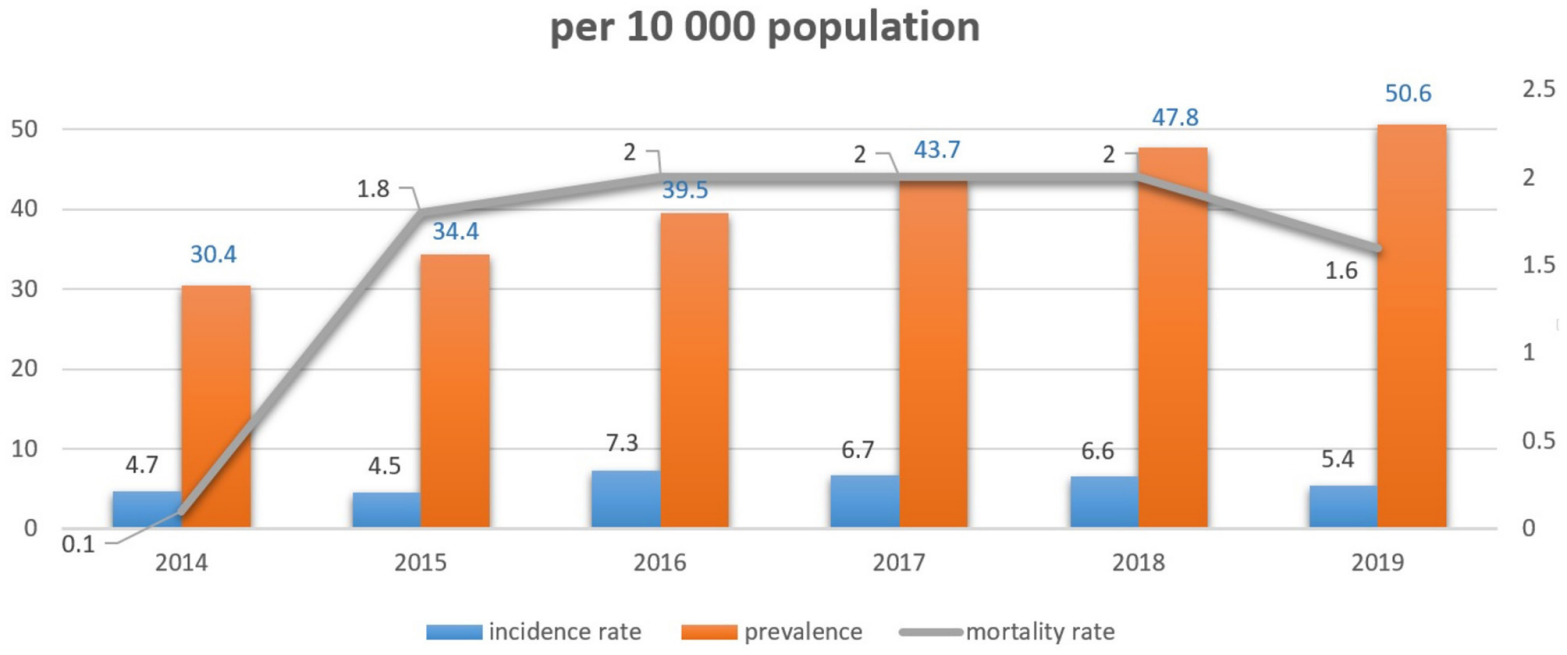
Incidence, prevalence and mortality rates of patients with breast cancer.

Incidence, prevalence, and crude mortality rates were assessed and are shown in Figure 2. Breast cancer incidence varied from 4.7 per 10,000 population in 2014 to 5.4 in 2019. The prevalence rate increased from 30.4 per 10,000 population in 2014 to 50.6 in 2019. At the same time, there was no obvious difference in breast cancer mortality for the observed period with a stable index of 2.0 per 10,000.

**Figure 3:**
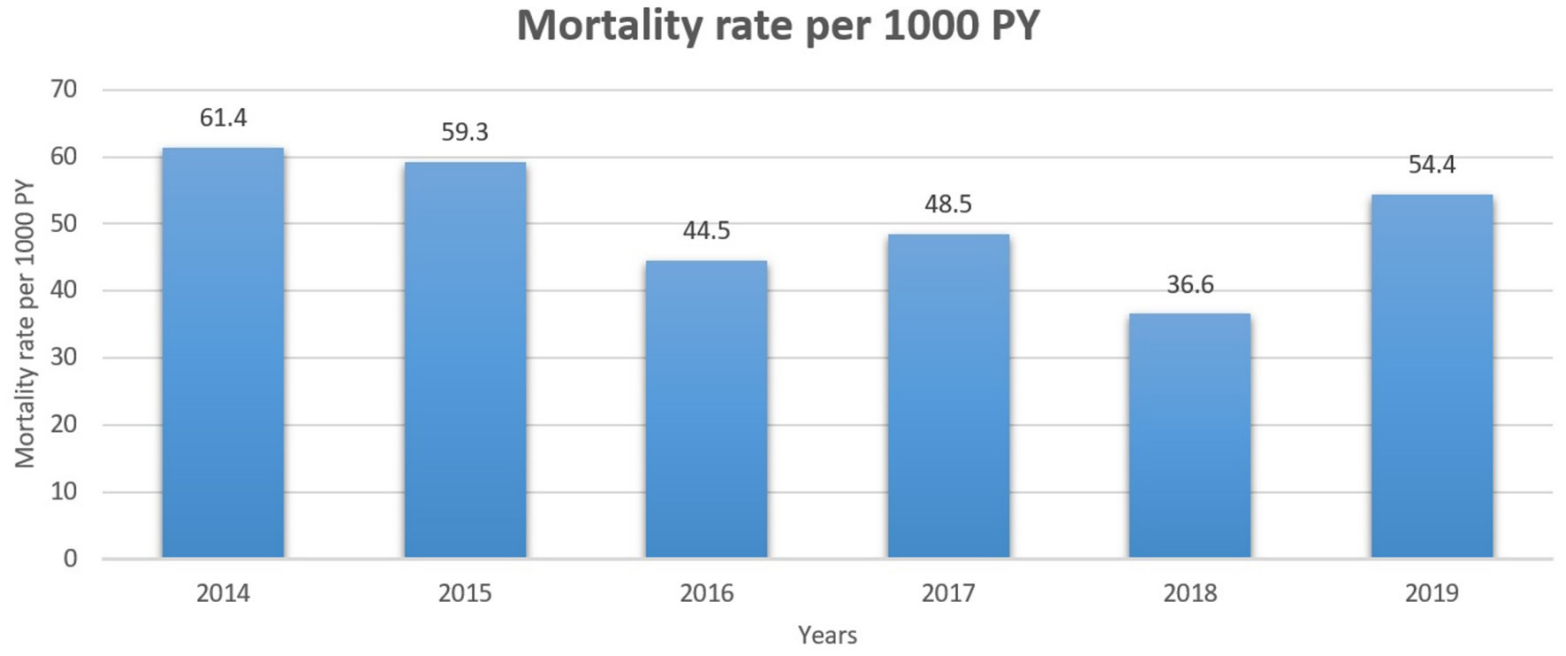
All-cause mortality rate of patients with breast cancer per 1000 person-years for 2014–2019 years.

The all-cause mortality rate of patients with breast cancer per 1,000 person-years is presented in Figure 3. Mortality rates varied from 61.4 per 1000 in 2014 to 54.4 in 2019.

**Figure 4:**
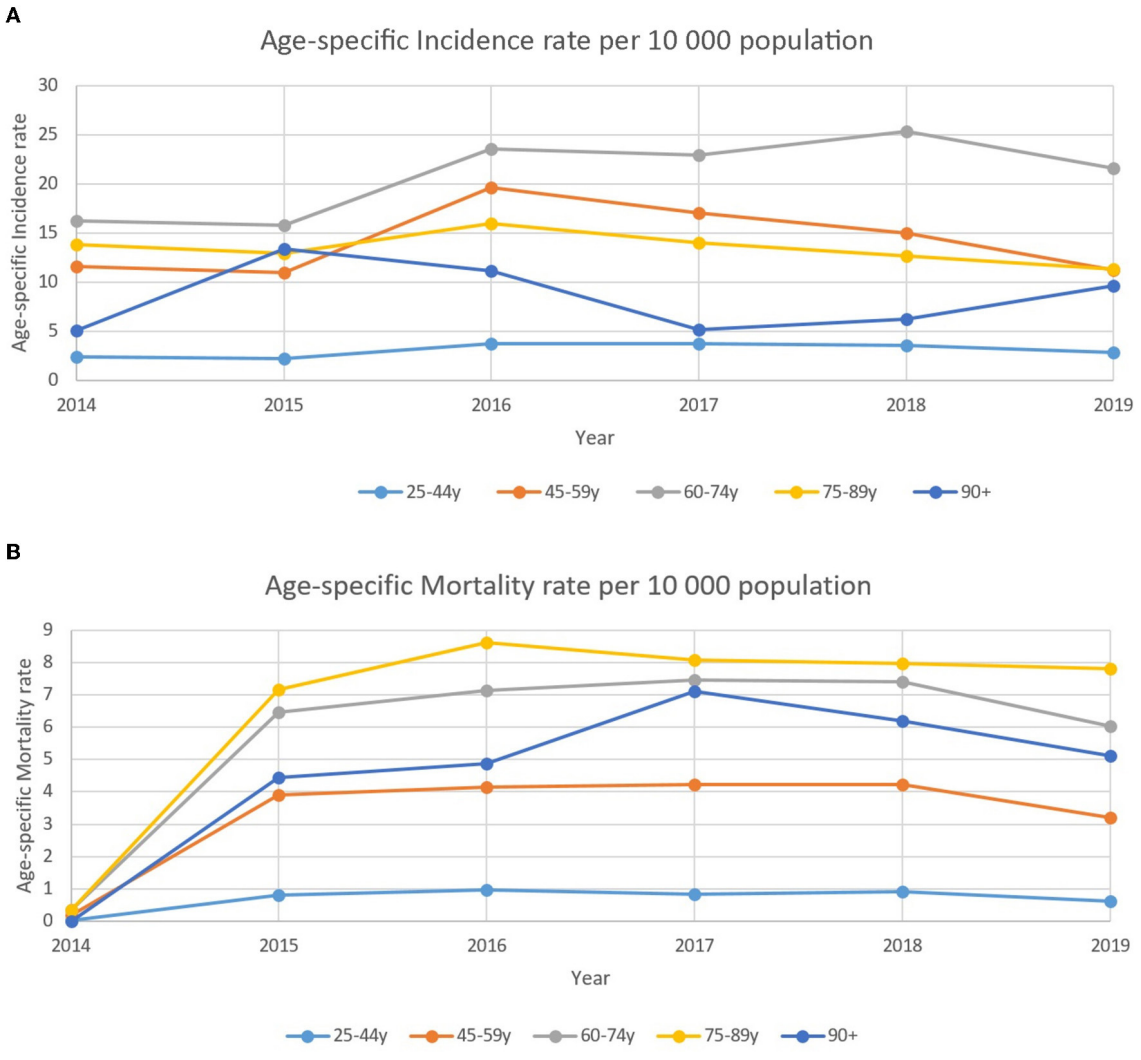
**(A)** Age-specific incidence rate of patients with breast cancer per 10,000 population between 2014–2019. **(B)** Age-specific standardized mortality rate of patients with breast cancer per 10,000 population between 2014–2019.

There were no major changes in age-specific incidence among breast cancer patients for the analyzed period of time with a stable and high incidence in the age group of 60–75 years with the highest rate of 25.3 per 10,000 in 2018 (Figure 4). At the same time, mortality rates did not show the same trend in the aforementioned group of patients. Standardized mortality rates were stable and high in senile age patients (75–89 years old).

Table 2 represents the association between socio-demographic and medical characteristics with all-cause mortality rates of breast cancer from 2014 to 2019. The Unadjusted Cox proportional hazard model shows that the older age group (>90) has the highest hazard of death (14.5) than other age groups. Slightly increasing trends can be observed in models adjusted to demographics and to comorbidities such as hypertension, diabetes, and stroke. Russian ethnic group had a 10% higher risk of death than other ethnic groups in the unadjusted model. However, in models adjusted to demographics and comorbidities, other ethnic groups showed a 10% higher risk of death. Patients with diabetes as a comorbidity had a 10% higher risk of death in the adjusted PH model.

**Table 2:** Association between socio-demographic and medical parameters and all-cause mortality rates of breast cancer between 2014 and 2019.

**Variable**	**Unadjusted**	***p*-value**	**Adjusted to demographics**	***p*-value**	**Adjusted to comorbidities**	***p*-value**
**Demographics**						
**Age groups**, ***n*** **(%)**						
25–44	ref.		ref.		ref.	
45–59	1.5 [1.4; 1.6]	<0.0001	1.6 [1.4; 1.7]	<0.0001	1.7 [1.6; 1.8]	<0.0001
60–74	2.9 [2.7; 3.1]	<0.0001	3.0 [2.8; 3.3]	<0.0001	3.4 [3.2; 3.7]	<0.0001
75–89	7.5 [6.9; 8.1]	<0.0001	7.9 [7.3; 8.6]	<0.0001	8.6 [7.9; 9.3]	<0.0001
>90	14.5 [10.7; 19.6]	<0.0001	15.7 [11.6; 21.3]	<0.0001	16.4 [12.1; 22.1]	<0.0001
**Ethnicity**, ***n*** **(%)**						
Kazakh	ref.		ref.		ref.	
Other	1.1 [1.0; 1.1]	0.014	1.2 [1.1; 1.3]	<0.0001	1.2 [1.1; 1.3]	<0.0001
Russian	1.2 [1.1; 1.2]	<0.0001	1.1 [1.0; 1.2]	<0.0001	1.1 [1.0; 1.2]	<0.0001
**Residence**, ***n*** **(%)**						
Urban	ref.		ref.		ref.	
Rural	1 [0.9; 1.1]	0.996	1.2 [1.1;1.4]	0.005	1.0 [0.9; 1.1]	0.985
**Comorbidities**						
Hypertension	0.5 [0.5; 0.6]	<0.0001			0.4 [0.4; 0.5]	<0.0001
Diabetes	1.1 [1.0; 1.1]	0.036			1.2 [1.1; 1.3]	<0.0001
Stroke	2.2 [2.0; 2.4]	<0.0001			2.0 [1.8; 2.2]	<0.0001

## 4. Discussion

This is the first in-depth epidemiological study in Kazakhstan and Central Asia, assessing the prevalence, incidence, and mortality rates of breast cancer using the Unified National Electronic Health System, 2014–2019.

We found that breast cancer incidence in Kazakhstan corresponds with worldwide trends and is increasing over the investigated period, while mortality rates slowly decline. This rise in incidence rate may be partially explained by alterations in lifestyle—such as later marriage, later first pregnancy, and fewer number of deliveries—the use of oral contraceptive pills, inactivity/obesity, and smoking. It is also conceivable that this growth is influenced in part by more accurate breast cancer detection and diagnosis. Breast cancer screening has been offered in Kazakhstan to women between 50 and 60 years old at a 2-year interval since 2008. Since 2014, 80% of mammography equipment was digitalized, and the screening age was raised to 40–70 years old.

The incidence rate of breast cancer for the study period among the female population in Kazakhstan varied from 4.5 to 7.2 cases per 10,000 persons. Age trends of breast cancer diagnosis vary slightly among the Asian and European countries; in Saudi Arabia, it is 55.68 years; in Iran, it is 46.76 ± 1.19 years, while in the USA, it is 63 years, and the global trend is 62 years old (17, 18). The average age of patients with BC in the republic of Kazakhstan during the study period was 55.7 ± 12.0 years and corresponds with the global trends.

Despite the average age of diagnosis in our study falling within 50–60 years, approximately 20% of our study population was younger and would not have been diagnosed with the screening program. It should be mentioned that the age of breast cancer patients is an important risk factor and prognostic factor. During the study period, 18.2% of patients were diagnosed with breast cancer at a young age (25–44 years). This category of patients does not fall under the criteria for mammographic screening in Kazakhstan which starts at the age of 40 (19). Data revealed that breast cancer is the most common cancer type in adolescents and young adults of age 15–39 years, accounting for one-third of all newly diagnosed cancer cases in young women. According to SEER, 5.6% of all invasive breast cancer cases were diagnosed in young women (6, 20). Young patients with breast cancer are more likely than older patients to present with advanced disease or aggressive biological tumor subtypes, such as triple-negative or HER2-positive breast cancer. Moreover, breast cancer in young patients was strongly associated with family history and genetic mutations in the BRCA1 or BRCA2 genes leading to the development of breast and ovarian cancers.

Young patients have a higher mortality rate than older patients, even among those with early-stage breast cancer (21). Young patients are also more susceptible to treatment-related adverse effects and cancer-related psychosocial problems. The medical community must pay particular attention to this reality, addressing these issues by drafting regulations and guidelines and establishing medical systems focused on early identification and prevention of breast cancer in young and adolescent patients (22).

In our study, 44.8% of patients diagnosed with breast cancer were patients belonging to 44–59 years with an average age of 57 ± 12.0 years, and this age is below the mean age of breast cancer diagnosis in other countries. The possible reason explaining this fact might be related to the best adherence to breast cancer screening among this group of patients. The fact that younger patients are more often diagnosed with aggressive tumor phenotypes may lead to their capture by the inpatient registry as they are receiving more aggressive treatment. Our study showed that 94% of all patients were from urban areas, and we explain this fact with better screening coverage of urban citizens in comparison with rural areas. The abovementioned groups of patients are an able-bodied population. Currently in Kazakhstan, like in other countries, jobs are increasingly moving out of agriculture into the urban services sector; at the same time, the urbanization rate will increase in Kazakhstan from 63 to 64% by 2050 (23). Among women aged from 45 to 64 years, breast cancer was commonly associated with higher work and home productivity days lost in the first 2 years since the diagnosis (24). As seen in other studies, productivity losses and potential losses in public finance associated with breast cancer in Europe increased to 20% in 2014 compared to 2010 (25). Islami et al. assessed lost profit as $6.2 billion due to premature mortality because of breast cancer among all age patients in 2015 (26). Further study should be done to assess the lost market earning due to breast cancer diagnosis among the Kazakhstan population. The peak of breast cancer incidence was at the age of 60–75 years, and it might correspond with the aging population in Kazakhstan. Data show that in 2021, nearly 8.17% of Kazakhstan's total population was aged 65 years and older, but in 2014, 6.76% were older than 65 years.

The majority of European countries reported that breast cancer was the leading cause of cancer-related deaths in 2018 with European Union breast cancer mortality rates from 17.9 in 2002 to 15.2 per 1,00,000 population in 2012 (1, 27, 28). Breast cancer shows the most prevalent malignancy and the second-largest cause of cancer-related deaths among Asian women (29). Across the coming 10 years, it is expected that the number of breast cancer-related deaths will rise in Asian nations. According to studies, Asia's mortality rates for breast cancer varied greatly in 2017 from 8.6 in East Asia to 15.0 per 1,00,000 patients in South Asia (30).

The mortality rate in breast cancer patients during the study period among the female population of the country did not show any negative or positive trend and varied from 1.8 to 2.0 per 10,000 patients and was the highest in senile patients, and it corresponds with available data. The study by Freedman R. showed that the risk of death over a 6-year follow-up period increased with age, counting 42% of women aged 75–84 years and 66% of women aged ≥85 years (31). While the relative survival rate of patients with advanced breast cancer has increased in recent years for those aged 65 to 75, there has been no improvement for those who are over 75 years (32).

As expected, patients who died were older on average (75–89 years), and it can be explained by the number of comorbidities. Many studies have reported that several comorbidities are associated with a lower survival rate among all breast cancer patients, and a high proportion of older patients die from non-cancer-related causes (31, 33, 34). All-cause mortality rates in our study varied from 61.4 per 1000 in 2014 to 54.4 in 2019. The most common comorbidity reported among breast cancer survivors in our study was hypertension (33.7%), followed by a history of surgery (12.9%), diabetes mellitus (11.3%), and stroke (1.9%).

Breast cancer mortality was positively associated in women who had been diagnosed with diabetes, 232 HR 1.2 (95% CI, 1.1–2.3), whereas it is negatively associated with arterial hypertension, HR 0.4 (95% CI, 0.4–0.5).

Cardiovascular disease is a known leading cause of non-cancer-related deaths in women who were diagnosed with breast cancer worldwide. The risk of cardiovascular disease is increased by both chemotherapy and radiotherapy. Different chemotherapy medications such as anthracyclines, fluoropirimidines, taxanes, and HER-2 targeted agents are associated with a high risk of cardiomyopathy, heart failure, as well coronary artery disease (35, 36). Vo et al. showed that patients who underwent previous breast cancer treatment in the US had a lower risk of heart disease and associated mortality in comparison with the general population. Those findings might be explained by national heart screening programs, improved healthcare access, and the expanding awareness of cardiovascular risk factors among healthcare providers and breast cancer patients (37). Our study also shows that more attention should be applied to clinical decision-making assistance in cancer survivors in order to manage late cardiovascular complications in this cohort of patients in Kazakhstan (38).

Our data correspond with the number of studies showing that diabetes increases the risk of breast cancer relapse and breast cancer-related deaths (39, 40). Diabetes patients in Sweden were shown to have a 45% higher chance of dying from breast cancer than non-diabetics in a hospital-based cohort study (39). There are different possible explanations for the impact of diabetes mellitus on the survival of patients with breast cancer. First, diabetes mellitus led to an increase in tumor cell proliferation rate which in turn can lead to an increased risk of breast cancer recurrence rate.

Breast cancer patients with diabetes mellitus developed insulin resistance and chronic hyperinsulinemia which might stimulate insulin receptor signaling and induce breast cancer cell proliferation and growth (41, 42). Diabetes mellitus as well as other comorbidities may decrease a patient's treatment options, leading to increased mortality due to a higher risk of treatment side effects (43).

It has to be emphasized that age-specific mortality rates were lower in patients who are older than 90 years compared to those who are 75–89 years old. This fact might be explained by the less aggressive tumor subtypes of that group of patients and the fact that significantly fewer patients who are older than 80 years underwent radiation therapy and chemotherapy as a part of their treatment (44, 45).

Our study had several advantages. In Kazakhstan, it is the first study to give comprehensive epidemiological data on incidence, prevalence, and mortality rates for breast cancer. Additionally, for a 6-year period, the whole female population of Kazakhstan was included in the study's big cohort (2014–2019). Data from medical records were connected to sociodemographic data and patient comorbidities.

However, this report does have several significant shortcomings. These issues stem from the UNEHS flaws, which were introduced in 2014 and are still being worked on. This system does not provide information on the patient's past medical history, past pregnancy and delivery history, education status, marital status, general family history, cancer disease stages, cancer molecular subtypes, and treatment given to patients. In this study, we have not had an opportunity for a detailed analysis of the breast cancer subtypes and stages as these data belong to the National Oncology Registry. It will, therefore, be a task for our subsequent investigations. The presence of these crucial variables could enhance the study's findings.

## 5. Conclusion

Breast cancer continues to be one of the leading causes of mortality in Kazakh women despite recent declines in incidence. To lower the death rate, it is crucial to continue developing evidence-based early identification policies and to optimize existing treatment approaches.

Breast cancer incidence in Kazakhstan is overall increasing, while mortality rates have begun to decline. The switch to population-based, high-quality mammography screening could reduce breast cancer mortality. These findings should be used to help Kazakhstan establish priorities for cancer control, including the need for the implementation of successful screening and prevention programs that are both cost-effective and efficient as well as the planning of future cancer services based on the allocation of limited resources to ensure their operationalization. Future research should be done to understand the role of effective management of comorbidities among breast cancer patients as an action to improve disease prognosis.

## Data availability statement

Dataset is not publicly available. Requests to access these datasets should be directed to Republican Center of Electronic Healthcare.

## Ethics statement

The study was approved by the NU Institutional Review Ethics Committee (651/24112022 on 28/11/2022), with an exemption from informed consent.

## Author contributions

AM, KM, and AG: conceptualization, methodology, validation, investigation, and writing—original draft preparation. KM and AG: software and formal analysis. KM, GZ, YS, and GR: data curation. AG, BS, DA, and OS: writing—review and editing. AM and KM: visualization. AG: supervision, project administration, and funding acquisition. All authors have read and agreed to the published version of the manuscript.
